# Supplementary figures and images for: Two distinct waves of transcriptome and translatome changes drive Drosophila germline stem cell differentiation
Source: EMBO J. 2024 Mar 13;43(8):10. doi: 10.1038/s44318-024-00070-z (PMC11021484; doi:10.1038/s44318-024-00070-z)

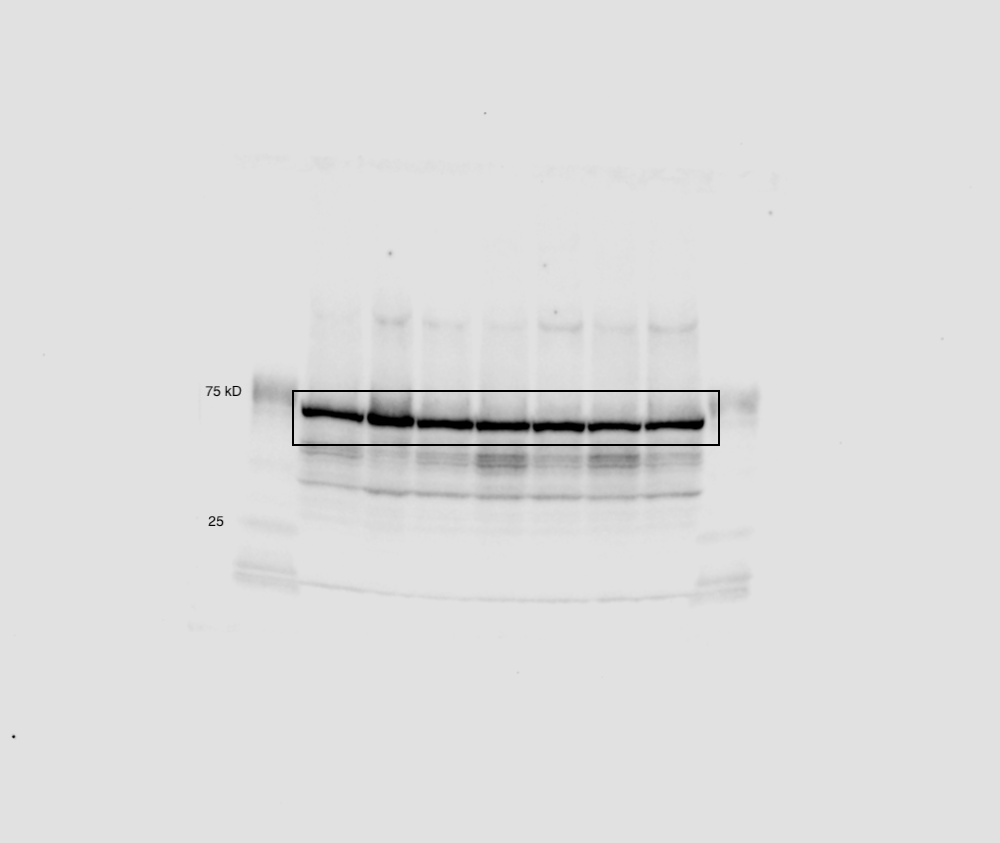

Supplement: Supplementary file 10 — Source Data Fig. 1 [file 44318_2024_70_MOESM10_ESM.zip › 1C/annotated_rabbit800_Vas_MP26.tif]

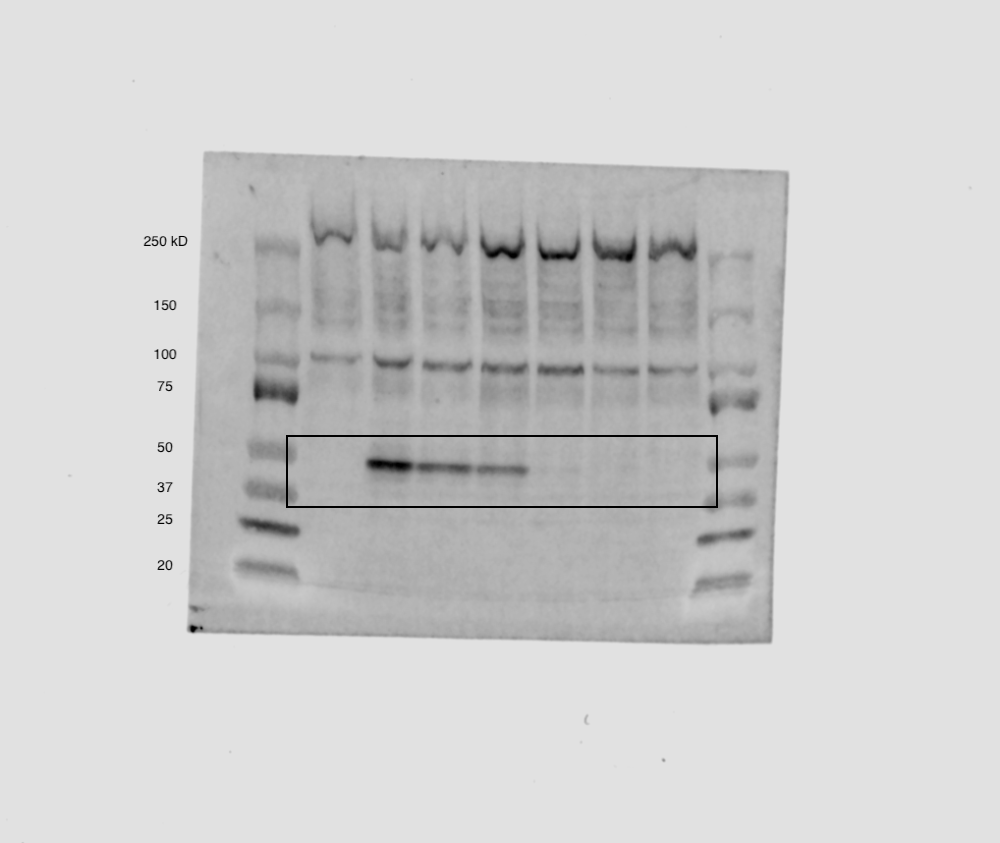

Supplement: Supplementary file 10 — Source Data Fig. 1 [file 44318_2024_70_MOESM10_ESM.zip › 1C/annotated_mouse680_Bam_aSpec_bCat_Orb_pH2Av.tif]
