# Supplementary material for: Two distinct waves of transcriptome and translatome changes drive Drosophila germline stem cell differentiation
Source: EMBO J. 2024 Mar 13;43(8):10. doi: 10.1038/s44318-024-00070-z (PMC11021484; doi:10.1038/s44318-024-00070-z)
Supplement: Supplementary file 14 — Expanded View Figures [file 44318_2024_70_MOESM14_ESM.pdf]

Expanded View Figures

Figure EV1. RNA-seq in synchronised differentiating GSCs recapitulates changes in gene expression during normal differentiation.

(**A**) As in Fig. 2A, smFISH in *wild type* germaria (right) was used to validate the RNA-seq results (left). Staining for DNA (Hoechst, blue), actin (phalloidin, gray), fusome (α-spectrin, yellow), and mRNA transcript of interest (smFISH, red and grayscale): (i) *cuff*, (ii) *thymidylate synthase*, (iii) *CG11674*, (iv) *CG32814 (eggplant)*, (v) *CG14545*, (vi) *blanks*. Scale bars are 15 µm. (**B**) Heatmap illustrating RNA-seq expression level across our time course, of marker genes identified by scRNA-seq pseudotime analysis by Rust et al, 2020 (illustrated in Fig. 2m in that paper, and approximate expression domain labelled here left) for each time point from GSC to 16cc. Each row represents one gene and expression level is scaled per gene: black— mean expression across the time course, gold—25% higher expression than the mean, cyan—25% lower expression than the mean. (**C**) The 0.5 h AHS sample was used to exclude 67 genes which showed a significant change of >threefold between the 0.5 AHS and the no HS samples (with expression >10 FPKM in one of these samples). The expression of these 67 genes is shown as log2foldchange at each time point relative to no HS. (**D**) Gene ontology enrichment analysis of the excluded 67 genes shown in (**C**) found a significant enrichment in terms associated with a heat shock response. Colour of the bar indicates *P* value of the enrichment. Background is the whole genome. GO enrichment analysis was performed using FlyMine (Lyne et al, 2007) with Holm–Bonferroni correction.

▶

© The Author(s)

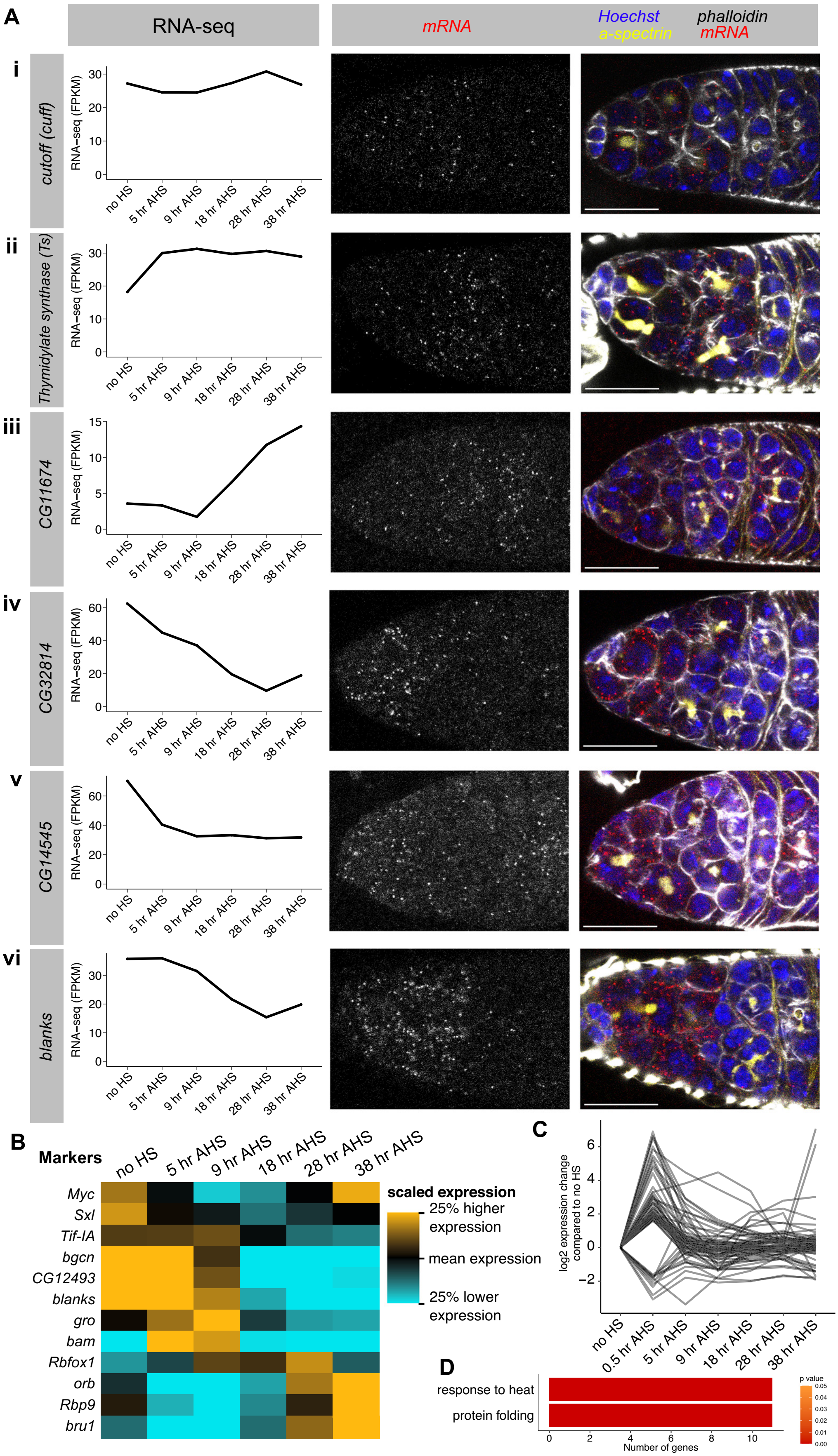
A
RNA-seq
mRNA
Hoechst
a-spectrin
phalloidin
mRNA
i
cutoff (cuff)
ii
Thymidylate synthase (Ts)
iii
CG11674
iv
CG32814
v
CG14545
vi
blanks
RNA-seq (FPKM)
no HS
5 hr AHS
9 hr AHS
18 hr AHS
28 hr AHS
38 hr AHS
B
Markers
no HS
5 hr AHS
9 hr AHS
18 hr AHS
28 hr AHS
38 hr AHS
Myc
Sxl
Tif-IA
bgcn
CG12493
blanks
gro
bam
Rbfox1
orb
Rbp9
bru1
scaled expression
25% higher expression
mean expression
25% lower expression
C
log2 expression change compared to no HS
no HS
0.5 hr AHS
5 hr AHS
9 hr AHS
18 hr AHS
28 hr AHS
38 hr AHS
D
response to heat
protein folding
Number of genes
p value

 © The Author(s)

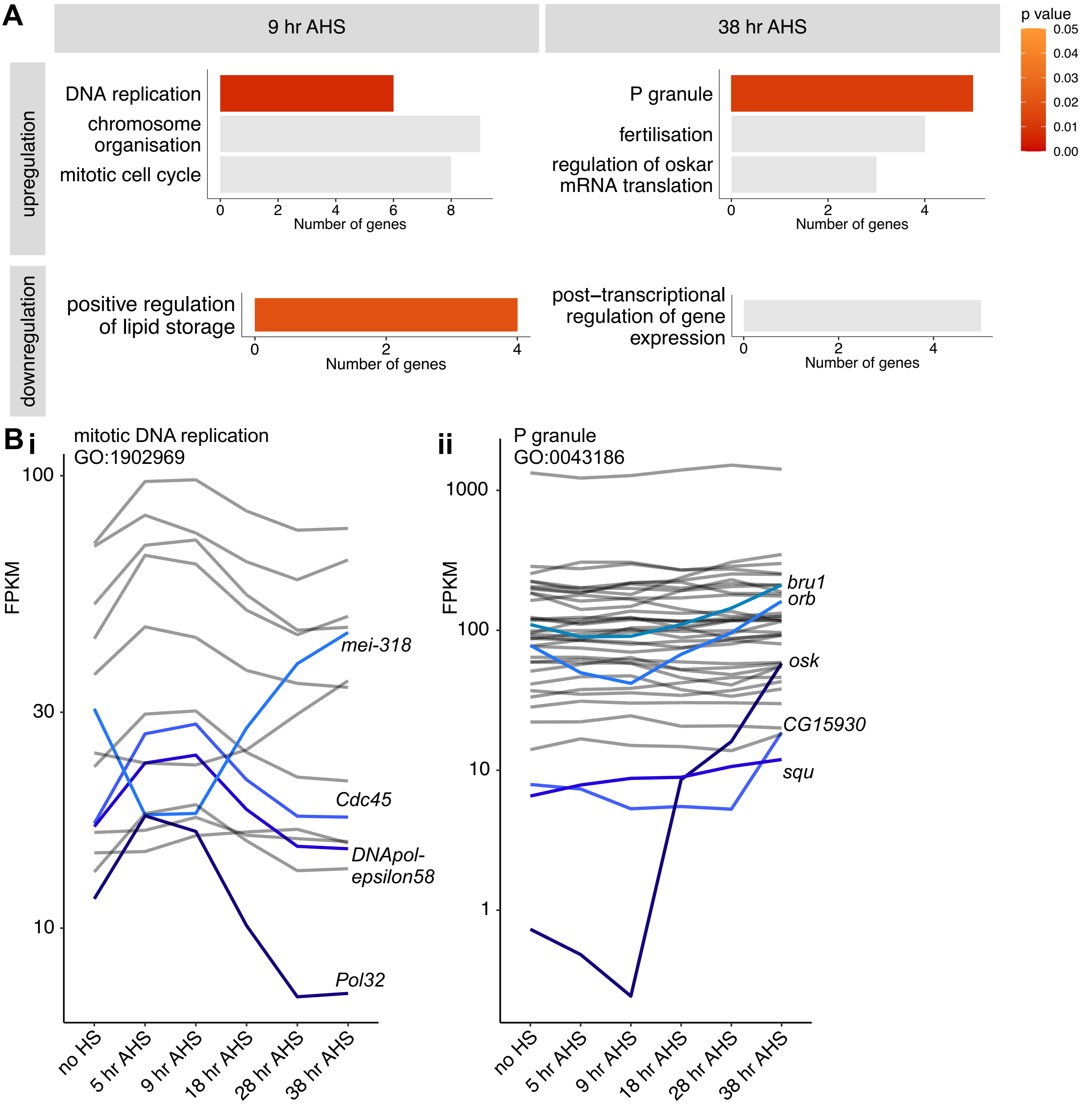

Figure EV2. RNA-seq reveals changes in mRNA level during differentiation.

(**A**) Gene ontology enrichment analysis of genes upregulated or downregulated in the RNA-seq at 9 h AHS or 38 h AHS compared to 'no HS'. Colour of the bar indicates p value of the enrichment, grey = $P > 0.05$. Background is all genes expressed during the time course. GO enrichment analysis was performed using FlyMine (Lyne et al, 2007) with Holm–Bonferroni correction. (**B**) RNA-seq FPKM for genes in two gene ontology groups: mitotic DNA replication (i) and P granule (ii). Genes with a >1.6-fold change in gene expression are highlighted.

© The Author(s)

A

Time point	Replicate R^2
no HS	0.9786
0.5 hr AHS	0.9831
5 hr AHS	0.9953
9 hr AHS	0.9891
18 hr AHS	0.9948
28 hr AHS	0.9761
38 hr AHS	0.9973

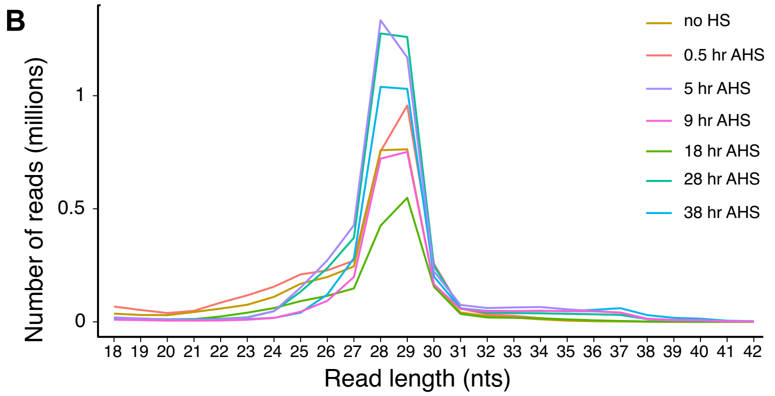

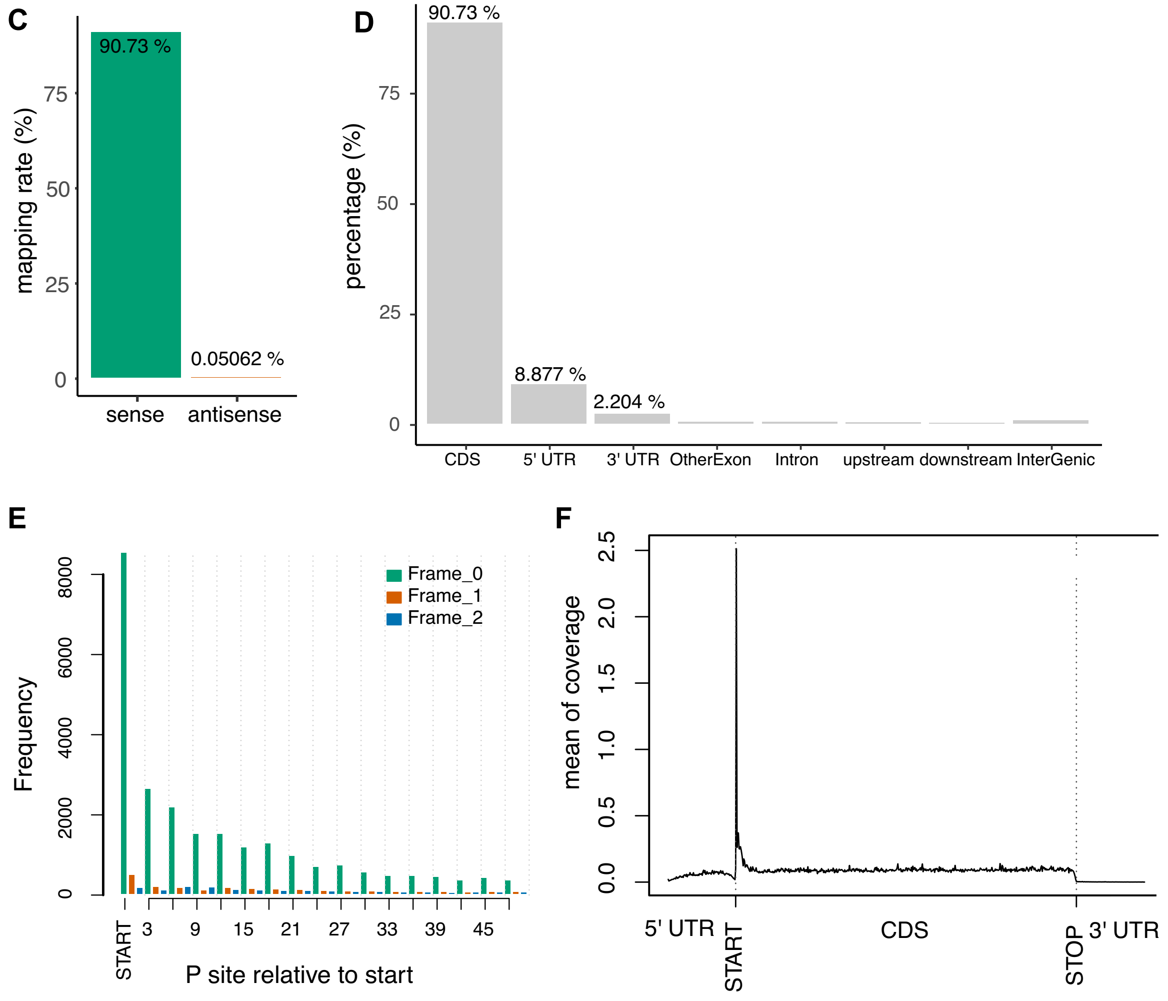

Figure EV3. Ribo-seq quality control.

(**A**) R^2 show good correlation between Ribo-seq replicate samples at each time point. (**B**) Distribution of read length in nucleotides for Ribo-seq libraries at each time point. (**C–F**) Ribo-seq quality control for the 'no HS' sample only for illustration. (**C**) 90.73% of reads mapped to the CDS on the sense strand of genes compared to 0.05% mapping to the CDS on the antisense strand. (**D**) 8.88% of reads map to the 5′ UTR, 2.20% map to the 3′ UTR, and mapping to introns and intergenic regions is negligible. (**E**) P-site mapping shows a strong three nucleotide periodicity, with highest frequency at the start codon. (**F**) Metagene analysis plot showing read distribution in 5′ UTR, CDS and 3′ UTR regions, shows consistent coverage across the CDS with the expected bias at the start codon.

 © The Author(s)

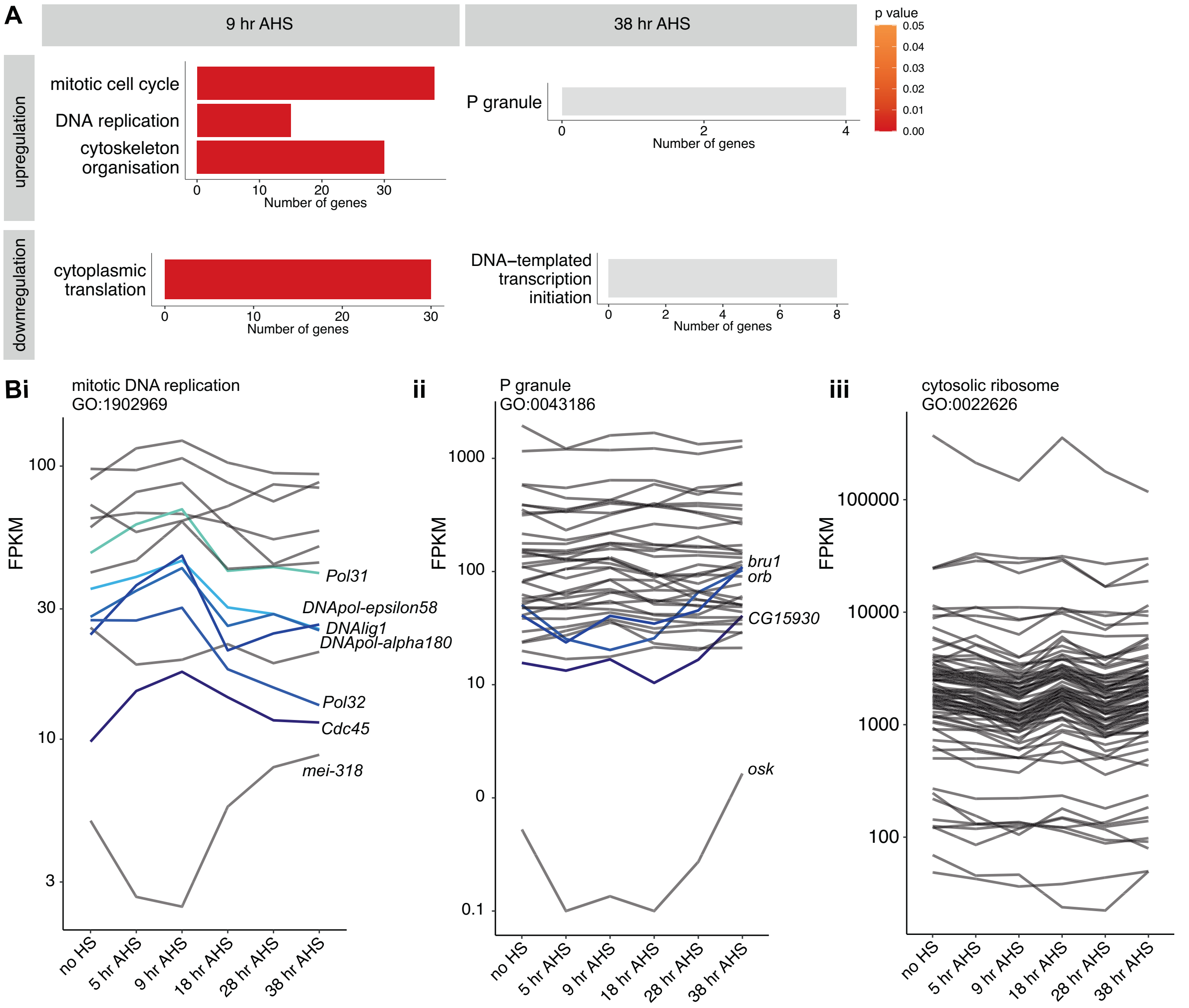

Figure EV4. Ribo-seq reveals changes in translation during GSC differentiation.

(**A**) Gene ontology enrichment analysis of genes upregulated or downregulated in the Ribo-seq at 9 h AHS or 38 h AHS compared to 'no HS'. Colour of the bar indicates p value of the enrichment, grey = $P > 0.05$. Background is all genes expressed during the time course. GO enrichment analysis was performed using FlyMine (Lyne et al, 2007) with Holm–Bonferroni correction. (**B**) Ribo-seq FPKM for genes in three gene ontology groups: mitotic DNA replication (i), P granule (ii) and cytosolic ribosome (iii). Genes with a >1.6-fold change in gene expression are highlighted (except in iii due to too many genes).

© The Author(s)

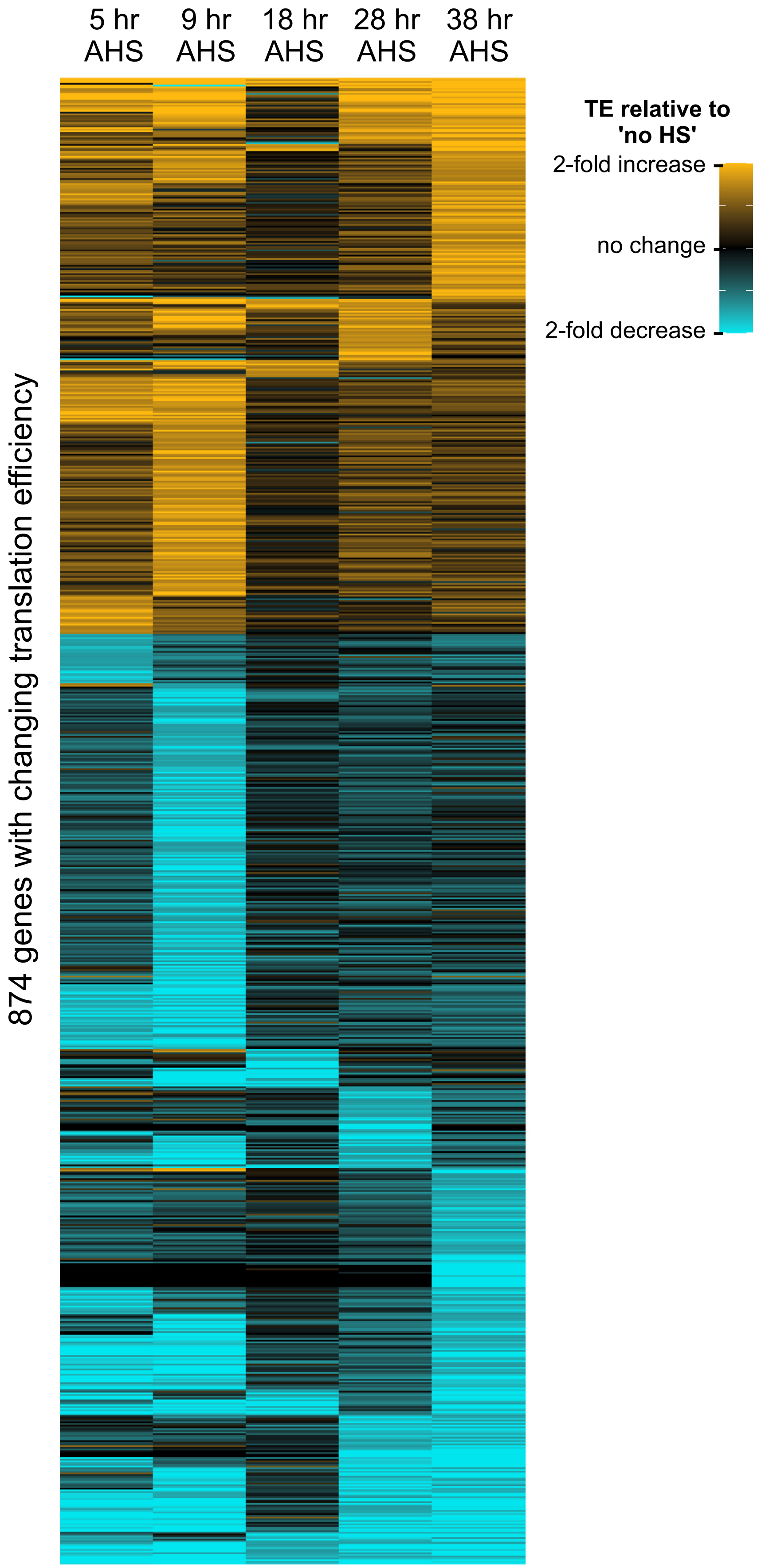
5 hr AHS
9 hr AHS
18 hr AHS
28 hr AHS
38 hr AHS
874 genes with changing translation efficiency
TE relative to 'no HS'
2-fold increase
no change
2-fold decrease

 © The Author(s)

Figure EV5. Many genes are regulated by TE.

Heatmap showing fold change in TE, compared to the 'no HS' time point, for the 874 genes which exhibit a significant 1.6-fold change in translation between a given time point and 'no HS'. cyan = twofold decrease compared to 'no HS', black = no change, gold = twofold increase.

© The Author(s)
